# Assessing intestinal permeability in Crohn’s disease patients using orally administered ^52^Cr-EDTA

**DOI:** 10.1371/journal.pone.0211973

**Published:** 2019-02-07

**Authors:** Julius Z. H. von Martels, Arno R. Bourgonje, Hermie J. M. Harmsen, Klaas Nico Faber, Gerard Dijkstra

**Affiliations:** 1 Department of Gastroenterology and Hepatology, University of Groningen, University Medical Center Groningen, Groningen, The Netherlands; 2 Department of Medical Microbiology, University of Groningen, University Medical Center Groningen, Groningen, The Netherlands; Texas A&M University College Station, UNITED STATES

## Abstract

**Background:**

Intestinal permeability can be assessed by monitoring renal excretion of orally administered radioactively ^51^Cr-labeled ethylenediaminetetraacetic acid (^51^Cr-EDTA). Although considered safe, patient participation in using radio-labeled tracers is low. Here, we used orally administered ^52^Cr-EDTA as non-radioactive alternative to assess intestinal permeability in CD and analyzed the association with disease activity, disease location and gut microbial dysbiosis.

**Materials and methods:**

60 CD patients with low (*n* = 25) and increased (*n* = 35) fecal calprotectin levels (cut-off: 100 μg/g feces) ingested 20 mL ^52^Cr-EDTA (20 mmol/L) solution whereafter 24-h urine was collected. Urinary ^52^Cr-EDTA concentrations were quantified using Inductively Coupled Plasma Mass Spectrometry (ICP-MS). Fecal *Enterobacteriaceae* and *Faecalibacterium prausnitzii* were quantified using FISH. Correlations between urinary ^52^Cr-EDTA excretion and other parameters were established using nonparametric Spearman’s correlation coefficients (*ρ*).

**Results:**

CD patients with increased fecal calprotectin levels (> 100 μg/g) demonstrated an elevated urinary ^52^Cr-EDTA/creatinine ratio (772 vs. 636 μmol/mol, *P* = 0.132). Patients with primarily colonic disease showed the highest ^52^Cr-EDTA excretion. Importantly, a positive correlation was observed for the urinary ^52^Cr-EDTA/creatinine ratio and fecal calprotectin levels (*ρ* = 0.325, *P* < 0.05). Finally, urinary ^52^Cr-EDTA/creatinine ratio negatively correlated with the relative abundance of *Faecalibacterium prausnitzii* (*ρ* = -0.221, *P* = 0.092), while positively correlating with *Enterobacteriaceae* (*ρ* = 0.202, *P* = 0.126).

**Conclusions:**

Orally administered and renal excreted ^52^Cr-EDTA may be used to assess intestinal permeability in CD and correlates with fecal calprotectin levels and bacterial species relevant to CD. This test may improve non-invasive detection of disease exacerbations and help monitor disease activity.

## Introduction

In Crohn’s disease (CD), a disturbed balance between the gut mucosal immune system and intestinal microbiota is intimately associated with diminished functioning of the intestinal barrier. This barrier is instrumental in the protection against adhesion and infiltration of intraluminal antigens into the underlying lamina propria, thereby maintaining a healthy gut. An important functional and measurable feature of the gastro-intestinal barrier constitutes intestinal permeability, which is influenced by both the gut microbiota and mucosal immunity. [1] Intestinal microbiota play a pivotal role in preserving gut epithelial barrier integrity. A strong link exists between microbial dysbiosis and intestinal permeability. [2] Intestinal dysbiosis in CD is characterized by increased numbers of (potentially) pathogenic bacteria, such as several members of the *Enterobacteriaceae*, and a decreased abundance of commensal bacteria, such as *Faecalibacterium prausnitzii*. Decreased abundancy of *Faecalibacterium prausnitzii* is consistently observed in CD and is associated with increased inflammation and presumably disrupted intestinal barrier integrity. [3,4] Similarly, *Enterobacteriaceae*, e.g. adherent-invasive *Escherichia coli* (AIEC), are known to possess multiple pathogenic mechanisms that can lead to loss of intestinal barrier function. [5] These typical shifts of many bacterial species that are characteristic for CD dysbiosis are putatively related to either gain or loss of intestinal epithelial barrier function (such as *Faecalibacterium prausnitzii* and *Enterobacteriaceae* (e.g. *Escherichia coli*), respectively). [6,7]

An impaired intestinal permeability has been associated with many gastro-intestinal (GI) and non-GI diseases. [1] In inflammatory bowel disease (IBD) research, there has been much scientific interest in unraveling the complex association between a comprised gut wall integrity and mucosal inflammation. Until now, however, it is not fully understood whether a defective mucosal barrier in IBD promotes intestinal inflammation or that impaired barrier function is secondary to the inflammatory process. [8,9] Nonetheless, the link between an increased intestinal permeability and increased inflammatory activity is well-established. [10] There is also increasing evidence that an increased intestinal permeability to macromolecules precedes the onset of IBD and/or is a sign of subclinical disease activity. It may therefore be used as an early predictor of clinical relapses in IBD patients. [11–14] This is illustrated by the fact that a subset of clinically healthy first-degree relatives of Crohn’s disease patients are genetically predisposed to an increased intestinal permeability as compared to healthy controls. [15,16]

Despite this strong association, inflammatory disease activity has hitherto been monitored inconsistently in clinical practice. Since a poor association exists between clinically active disease and observed endoscopic disease activity, non-invasive biomarkers have increasingly been studied to predict active intestinal inflammation in CD. [17] For instance, biochemical disease parameters such as the fecal calprotectin (FC) level and serum C-reactive protein (CRP) are being widely utilized, since these markers correlate significantly with endoscopically active disease. [18–20] In this study, we use fecal calprotectin levels as an accurate indirect measure of inflammatory disease activity. [21,22]

Currently, orally administered tracers for intestinal permeability play no significant role in monitoring disease exacerbations in CD, but may aid in the diagnostic accuracy of the currently available panel of non-invasive disease biomarkers and help predict CD disease course. [19,23,24] The clinical utility and validity of these non-invasive tests for intestinal permeability are still not fully established. [1] An optimal intestinal permeability test should have a number of properties. The test or marker is preferably sensitive, accurate, reproducible and safe in order to assist in diagnosis and monitoring of disease flares. In addition, an *in vivo* marker should be stable and inert, avoiding metabolism and degradation. [1,14]

Numerous studies have evaluated intestinal permeability using radioactively ^51^Cr-labeled ethylenediaminetetraacetic acid (^51^Cr-EDTA). [11,12,25–33] However, these previously published studies have been unable to establish a consistent correlation between ^51^Cr-EDTA-measured gut permeability and inflammatory disease activity, likely related to relatively small and heterogeneous IBD study cohorts. Apart from this, most of these studies used the less preferred radioactively ^51^Cr-labeled EDTA, followed by urinary analysis of gamma radiation. Conjointly, its individual performance in clinical practice remains mostly unfeasible due to complex and impractical detection methods. [1,29] A similar substance has recently been developed to evaluate intestinal permeability, in which the non-radioactive ^52^Cr isotope has been incorporated into ^52^Cr-EDTA. This allows us to measure urinary ^52^Cr content in a safe, inexpensive and precisely validated manner using the highly sensitive method of Inductively Coupled Plasma Mass Spectrometry (ICP-MS). In contrast to radioactively labeled ^51^Cr-EDTA, only few studies have evaluated the performance of ^52^Cr-EDTA as a tracer for intestinal permeability. [34–36] Cr-EDTA has proven to be a sensitive marker for human intestinal permeability, both in health and disease. [35–39] Cr and EDTA form a stable and inert complex with one of the highest affinity of known metals, without any physicochemical interactions that might be expected to occur. [40,41] However, putative correlations between ^52^Cr-EDTA-measured intestinal permeability and CD-specific disease parameters have not yet been established.

The aim of this study is to evaluate intestinal permeability through measuring 24-h urinary excretion of orally administered ^52^Cr-EDTA in Crohn’s disease and its association with inflammatory disease activity, disease localization and two key bacterial marker strains of CD dysbiosis (*Faecalibacterium prausnitzii* and *Enterobacteriaceae*).

## Materials and methods

### Study population

Patients aged 18–65 years were recruited from March 2016 until April 2017 at the outpatient IBD clinic of the University Medical Center Groningen (UMCG), Groningen, The Netherlands. All patients were diagnosed with Crohn’s disease (CD), according to clinical, endoscopic and histopathological features. All patients were Caucasians and lived in the Northern part of the Netherlands. Patients were divided into two groups based on fecal calprotectin levels as a measure of inflammatory disease activity (using 100 μg calprotectin / gram feces as our currently used cut-off value for defining low and increased inflammatory disease activity). Standard demographic characteristics, including age, sex, body-mass index (BMI) and smoking history, were recorded, as well as clinical parameters specific for CD, such as the Harvey-Bradshaw index (HBI), Montreal disease classification (including Age at diagnosis (A), Location of disease (L) and the Behavior (B) of the disease) and current maintenance therapy. Disease location was recorded from the most recently performed endoscopic evaluation, which was completed within 12 months of intestinal permeability testing. CD patients who were treated with antibiotics 3 months prior to screening were not eligible for this study (to rule out an effect on the microbiota composition). Furthermore, CD patients with severe disease activity, as reflected by a HBI score > 12, or patients with an indication for remission-induction therapy, were also excluded from this study. Routine laboratory examinations were performed, including hemoglobin, C-reactive protein (CRP), erythrocyte sedimentation rate (ESR), leukocyte count, thrombocyte count and creatinine levels. Fecal calprotectin levels were quantified by enzyme-linked immunosorbent assays (ELISA) (BÜHLMANN Laboratories AG, Switzerland) as a routine measurement in the UMCG. Samples were obtained after patients gave written informed consent. This study has been approved by the Institutional Medical Ethical Review Board (in Dutch: Medisch Ethische Toetsingcommissie, METc) of the University Medical Center Groningen (UMC Groningen) (IRB no. 2014/291) and is in accordance with the principles of the Declaration of Helsinki (2013).

### ^52^Cr-EDTA solution

The chromium-EDTA oral test solution consists of the active ingredients chromium(III)chloride (CrCl_3_.6H_2_O) and disodium edetate (C_10_H_14_N_2_Na_2_OH.2H_2_O), 70% sorbitol liquid crystalline and raspberry essence as sweeteners, 0.1 M NaOH for pH adjustment to reach a final pH between 4.5–5.5, methylparahydroxybenzoate as preservative and purified water (according to Ph. Eur. requirements) as solvent. The solution contained in total 400 μmol of ^52^Cr-EDTA (20 mmol/L). Patients were instructed to drink the ^52^Cr-EDTA solution (20 mL) together with a glass of water after an overnight fast. Subsequently, patients were asked to fast for an additional 2 hours. Urine was collected for 24 hours from the moment of ingestion of the ^52^Cr-EDTA test solution. Patients were also instructed to refrain from the intake of alcoholic beverages or non-steroidal anti-inflammatory drugs (NSAID’s) two days prior to taking the ^52^Cr-EDTA solution and during the 24-h urine collection period. Full details on the preparation of the ^52^Cr-EDTA solution were described previously. [42]

### Analytical procedure

Urinary content of ^52^Cr was quantified using Inductively Coupled Plasma Mass Spectrometry (ICP-MS), which is a highly sensitive analytical method for measuring various metals, including chromium isotopes (Nexion 300X, Perkin Elmer, Medlon BV Enschede, The Netherlands). [43] First, 24-h urine samples were 30-fold diluted with 0.5% HNO_3_ and 0.01% Triton X-100, after which ^103^Rh was added as internal standard (IS). Subsequently, the sample analyte was nebulized in a cyclonic spray chamber, atomized at extremely high temperature (ranging from 6,000–8,000°C) and ionized using argon (Ar) plasma. Using a vacuum of ~ 20 mL/min, the resulting ions were deflected into a quadrupole in which ions are separated based on their electrical charge and mass, under the influence of an alternating electrical current. Finally, chromium ions were detected via an electron multiplier. Urinary chromium (^52^Cr) was measured while applying the kinetic energy discrimination (KED) mode, ensuring a general reduction of polyatomic isobaric interferences (such as that of ^40^Ar^12^C). Final urinary ^52^Cr concentrations (nmol/L) were divided by urinary creatinine concentrations (mmol/L) as correction for hydration. Urinary creatinine levels were quantified by an enzymatic detection method following supplier’s instructions (Roche Diagnostics, Roche Modular P Analyzer, Mannheim, Germany).

### Quantification of fecal bacteria using fluorescent in-situ hybridization (FISH)

The quantification of *Faecalibacterium prausnitzii* and *Enterobacteriaceae* was performed as previously described, with some minor modifications. [44] Patients were asked to provide fecal samples at the time of the 24-h urine collection. Samples were mixed with 4.5 mL filtered phosphate-buffered saline (PBS) and centrifuged at 700 *g* for 2–3 min. The resulting supernatant was 4-fold diluted with freshly-prepared 4% paraformaldehyde solution and stored overnight at 4°C. Before counting, samples were coded and randomized by an independent investigator. Serial dilutions were prepared to allow visual counting of total bacteria, *Enterobacteriaceae* and *F*. *prausnitzii*. Each dilution was spread over gelatin-coated glass slides and dried at room temperature. After addition of the appropriate bacterial probes (see **Table 1**), Eub338 (Rhodamine) for the total bacteria, Fprau645 (FITC) for *F*. *prausnitzii* and Ec1531 (CY3) for *Enterobacteriaceae*, slides were hybridized overnight at 50°C. [45–47] In each glass slide well, 25 fields were manually quantified using fluorescent microscopes (Leica 2 or Olympus BH20 at 100X magnification). Bacteria were quantified using two different filters, FITC or CY3, based on the probe colour. Based on the absolute bacterial counts, the relative percentage of each bacterial group was calculated.

**Table 1:** Bacterial probes used in the fluorescent in-situ hybridization (FISH).

Target	Probe	Label	Sequence 3’ > 5’
**Total bacteria**	Eub338	Rhodamine	*TGAGGATGCCCTCCGTCG*
***F*. *prausnitzii***	Fprau645	FITC	*CAAAAAGAACTCATCACGTCTCC*
***Enterobacteriaceae***	Ec1531	CY3	*ACTACTGCTCCGTGATGCCAC*

### Statistics

Characteristics of the study population were shown as proportions (%, *n*), means ± standard deviations (SD) or medians with interquartile range (IQR), as appropriate. Normality testing was performed using Kolmogorov-Smirnov tests. Distributions of urinary ^52^Cr-EDTA/Cr excretion were presented as median ± interquartile ranges (IQR) and presented in boxplots (10^th^-90^th^ percentiles) grouped by inflammatory disease activity, as determined by the fecal calprotectin level (using 100 μg/g feces as cut-off value). Differences between groups were tested using the independent sample *t*-test or Mann-Whitney U-test, as appropriate. Partial correlations between urinary ^52^Cr-EDTA/creatinine ratio and other parameters were performed using the nonparametric Spearman’s correlation coefficient (*ρ*). Associations between correlated parameters were visualized in scatter plots with smoothed curves. Smoothing was empirically applied by nonlinear regression using 2^nd^ order polynomial functions with 1/y^2^ weighting. Statistical analyses were performed using SPSS Statistics 23.0 for Windows. *P*-values ≤ 0.05 were considered as statistically significant.

## Results

Study cohort characteristics are shown in **Table 2**. Crohn’s disease (CD) patients with low fecal calprotectin levels (*n* = 25) had a mean age of 44.1 ± 13.3 years and consisted of 8 males (32.0%) and 17 females (68.0%), while patients with increased fecal calprotectin levels (*n* = 35) had a mean age of 40.5 ± 12.2 years and consisted of 10 males (28.6%) and 25 females (71.4%). Patients with increased levels of fecal calprotectin showed significantly elevated white blood cell counts (WBC) (*P* < 0.05) and erythrocyte sedimentation rates (ESR) (*P* < 0.05). CD patients with an increased level of fecal calprotectin had a significantly lower alcohol consumption as compared to CD patients with low fecal calprotectin levels (*P* < 0.01). No significant differences were observed for any other cohort characteristic. Also, clinical disease activity, as measured by the Harvey-Bradshaw Index (HBI), was not significantly different between patients with either low or increased fecal calprotectin levels. In the total study cohort, median HBI score was 3 (IQR: 1–5), indicating that the majority of patients were in clinical remission at the time of inclusion.

**Table 2:** Characteristics of Crohn’s disease patients (*n* = 60) with fecal calprotectin levels indicative of remissive disease (< 100 μg/g) and increased disease activity (> 100 μg/g). Data are presented as numbers (*n* (%)), mean ± SD or median [IQR]^†^.

**Characteristics**	**FC < 100 μg/g (*n* = 25)**	**FC > 100 μg/g (*n* = 35)**	***P-*value**
**Age (years)**	44.1 ± 13.3	40.5 ± 12.2	0.431
**Male gender**	8 (32.0)	10 (28.6)	0.783
**BMI (kg/m^2^)**	25.6 ± 4.5	24.3 ± 5.4	0.233
**Active smoking**	5 (20.0)	7 (20.0)	1.000
**Alcohol consumption (g/day)**	3.6 [0.9;11.1]	0.8 [0.1;2.6]	0.009**
**Ileocecal resection**	11 (44.0)	13 (37.1)	0.606
**HBI**			0.313
**Remission (< 5)**	17 (68.0)	25 (71.4)	
**Mild disease (5–7)**	6 (24.0)	4 (11.4)	
**Moderate disease (8–12)**	2 (8.0)	6 (17.1)	
**Maintenance medication**			0.936
**None**	7 (28.0)	8 (22.9)	
**Thiopurines**	5 (20.0)	9 (25.7)	
**Mesalamine**	3 (12.0)	6 (17.1)	
**TNF-antagonists**	7 (28.0)	9 (25.7)	
**Combination therapy**	3 (12.0)	3 (8.6)	
**Montreal,** **Age at diagnosis**			0.397
**A1 (< 17 yr)**	4 (16.0)	5 (14.3)	
**A2 (17–40 yr)**	15 (60.0)	26 (74.3)	
**A3 (> 40 yr)**	6 (24.0)	4 (11.4)	
**Montreal, Localization**			0.476
**L1 (ileal)**	12 (48.0)	12 (34.3)	
**L2 (colonic)**	4 (16.0)	5 (14.3)	
**L3 (ileocolonic)**	9 (36.0)	18 (51.4)	
**Montreal, Behavior**			0.642
**B1 (non-stricturing, non-penetrating)**	11 (44.0)	18 (51.4)	
**B2 (stricturing)**	9 (36.0)	13 (37.1)	
**B3 (penetrating)**	5 (20.0)	4 (11.4)	
**Laboratory values**			
**CRP (mg/L)**	0.9 [0.5;3.3]	2.6 [0.9;5.0]	0.052
**ESR (mm/h)**	8.0 [3.0;18.0]	13.0 [9.0;27.0]	0.028*
**WBC (x10^9^/l)**	6.4 ± 1.6	7.8 ± 2.2	0.013*
**FC (μg/g)**	45 [40;67]	360 [175;760]	0.000***
**Thrombocytes (x10^9^/l)**	288 ± 82	287 ± 67	0.776
**Creatinine (μmol/l)**	75 ± 15	71 ± 11	0.245

^†^FC, fecal calprotectin; BMI, body mass index; HBI, Harvey Bradshaw Index; TNF, tumor necrosis factor; CRP, C-reactive protein; ESR, erythrocyte sedimentation rate; WBC, white blood cell count. Differences between groups were tested using the independent sample *t*-test or Mann-Whitney U-test for continuous variables and the Fisher’s exact test for discontinuous variables, as appropriate. Two-sided *P*-values < 0.05 were considered as statistically significant.

*P < 0.05

**P < 0.01

***P < 0.001.

### Urinary ^52^Cr-EDTA/creatinine excretion proportionally increases with fecal calprotectin levels

**Fig 1:**
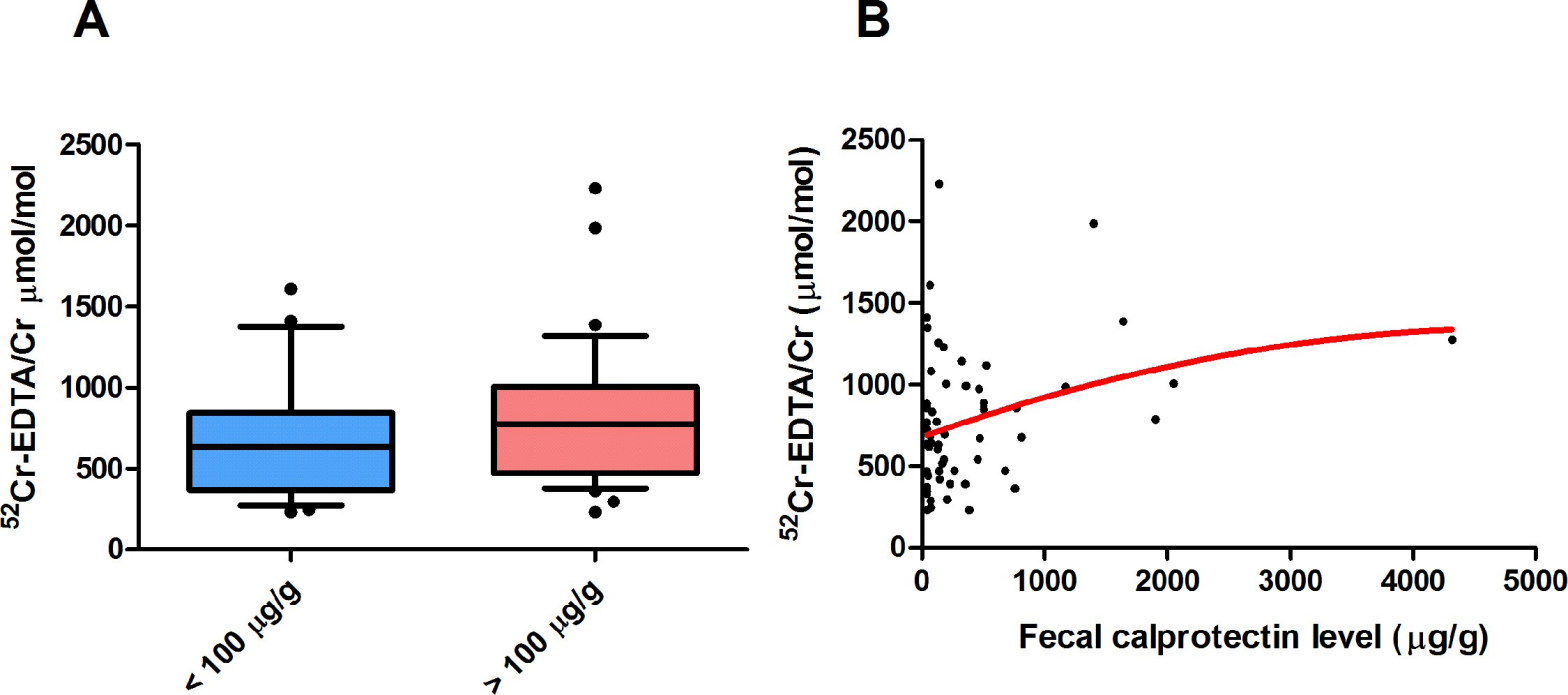
The relationship between levels of urinary 24-h ^52^Cr-EDTA/creatinine excretion and fecal calprotectin. **(A)** Distribution of urinary ^52^Cr-EDTA/creatinine excretion in patients with low (blue, < 100 μg/g) and increased (red, > 100 μg/g) fecal calprotectin levels, shown in boxplots. **(B)** Urinary ^52^Cr-EDTA/creatinine excretion significantly correlates with fecal calprotectin levels (*ρ* = 0.325, *P* < 0.05), as represented by the red smoothed curve.

Distributions of the 24-h urinary ^52^Cr-EDTA/creatinine ratio among both groups are shown in **Fig 1A**. CD patients with increased fecal calprotectin levels showed a trend of elevated urine-excreted ^52^Cr-EDTA/creatinine (771.8 μmol/mol (IQR: 472.4–1005.9) vs. 636.4 μmol/mol (IQR: 365.0–843.4), *P* = 0.132). Importantly, we observed a significant correlation between urinary ^52^Cr-EDTA/creatinine excretion and fecal calprotectin levels (*ρ* = 0.325, *P* < 0.05, **Fig 1B**), while controlling for gender, disease localization, ileocecal resection status and alcohol consumption.

### Urinary ^52^Cr-EDTA/creatinine excretion is highest in CD patients with solely colonic disease

**Fig 2:**
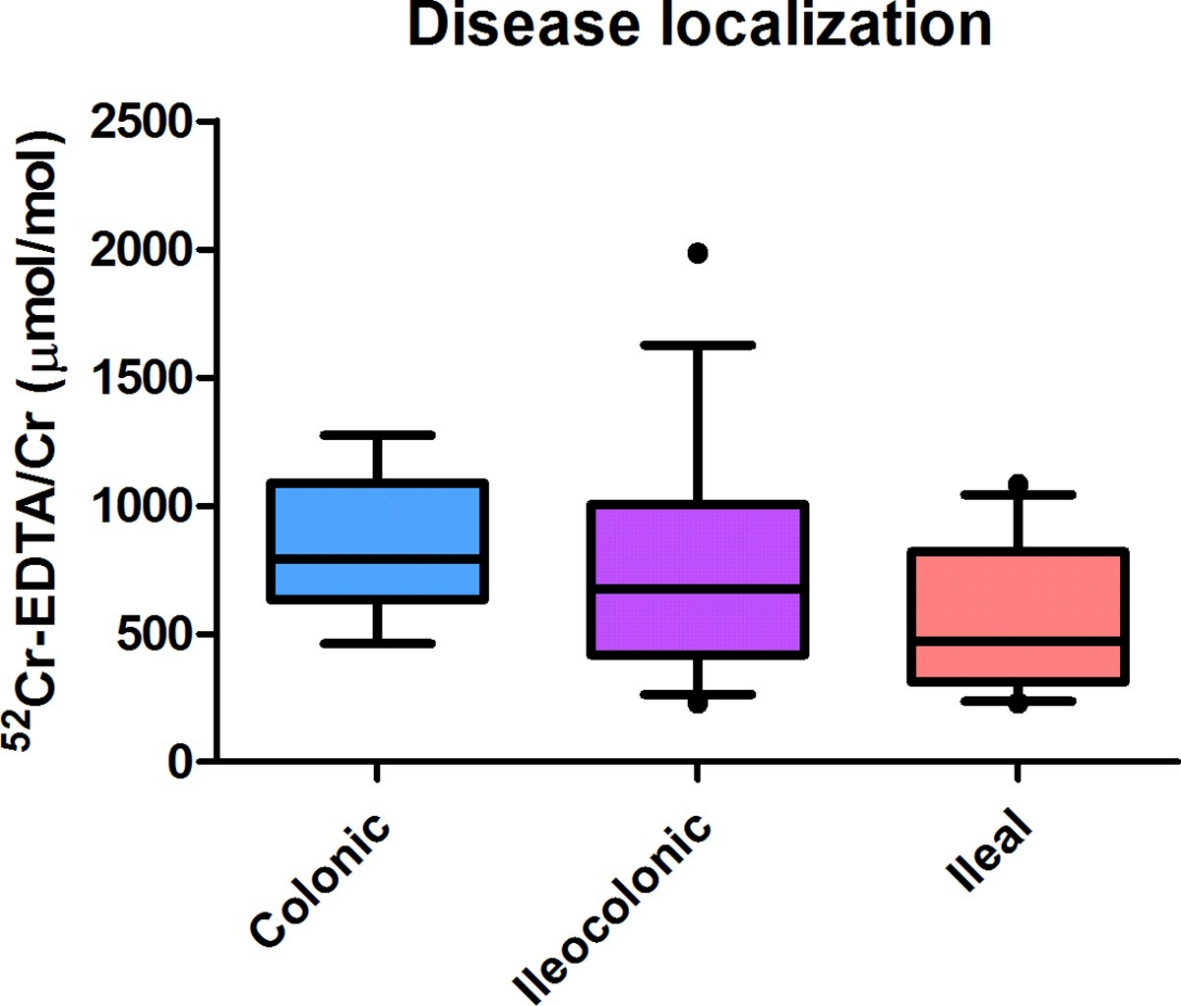
Urinary 24-h excretion of ^52^Cr-EDTA/creatinine for different subgroups of CD patients according to the Montreal classification of disease localization. No significant differences were observed between any groups (*P* > 0.05).

No significant differences in 24-h urinary excretion of ^52^Cr-EDTA/creatinine were observed for subgroups of CD patients with different disease localization according to the Montreal classification (*P* = 0.270, **Fig 2**). Still, CD patients with solely colonic disease showed the highest median urinary ^52^Cr-EDTA/creatinine excretion (791 μmol/mol (IQR: 632–1088), *n* = 9), whereas median excretion in patients with ileal involvement was lower (ileal disease: 472 μmol/mol (IQR: 312–821), *n* = 24; ileocolonic disease: 676 μmol/mol (IQR: 417–1006), *n* = 27, *P* = 0.093).

### Correlation analysis between urinary ^52^Cr-EDTA/creatinine excretion and relative abundances of CD-associated bacterial species

**Fig 3:**
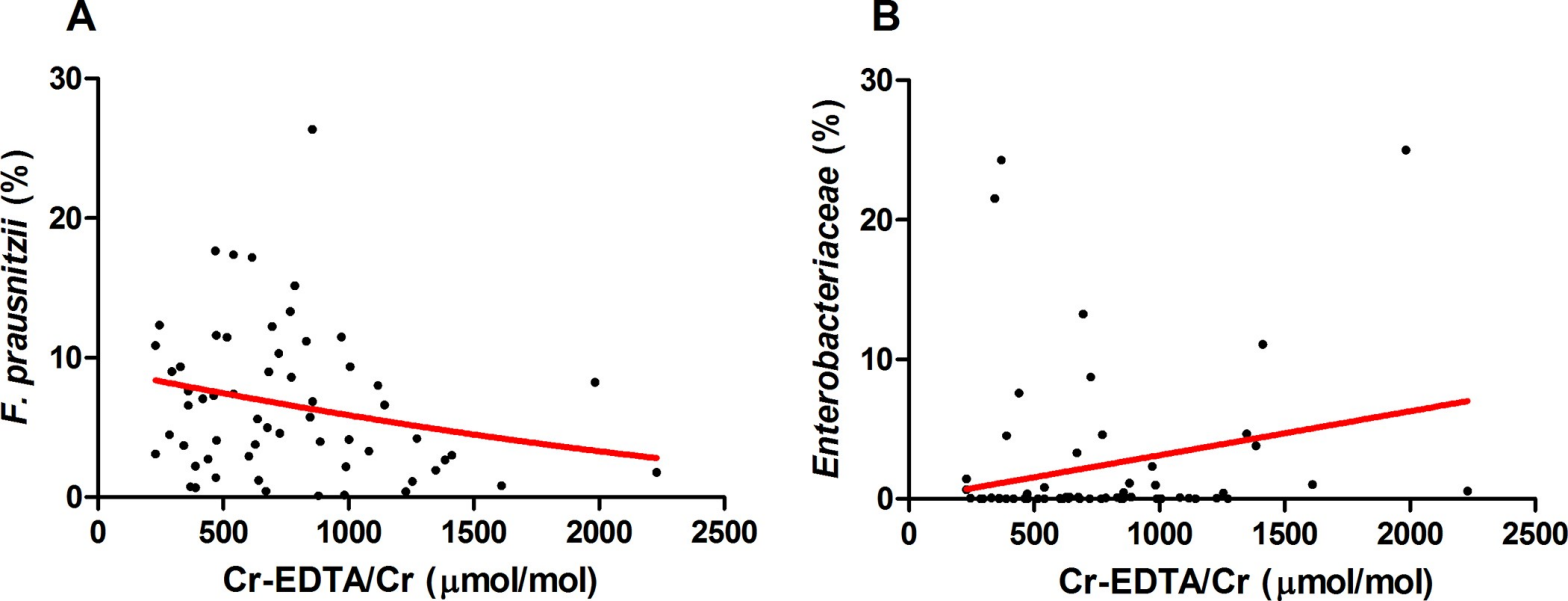
Correlations of 24-h urinary excretion of ^52^Cr-EDTA/creatinine with the relative abundances of key bacterial species in the diseased gut, as represented by the red smoothed lines. **(A)** A negative correlation (*ρ* = -0.221, *P* = 0.092) is observed between the relative abundance (%) of *F*. *prausnitzii* and urinary ^52^Cr-EDTA/creatinine excretion, though this was not statistically significant. **(B)** A positive correlation (*ρ* = 0.202, *P* = 0.126) is observed between the relative abundance (%) of *Enterobacteriaceae* and urinary ^52^Cr-EDTA/creatinine excretion, though this correlation was also not statistically significant.

The absolute and relative abundance of two CD-associated bacteria, *F*. *prausnitzii* and *Enterobacteriaceae*, was quantified by FISH analysis in fecal samples of all patients (see **S1 Fig** for representative fluorescence microscopy images and **S1 Table** for quantifications). Among CD patients with below-median and above-median ^52^Cr-EDTA/creatinine excretion, there were no significant differences detected in relative abundances (%) of *F*. *prausnitzii* and *Enterobacteriaceae*. Interestingly, we observed a borderline non-significant negative correlation between 24-h urinary ^52^Cr-EDTA/creatinine excretion and relative numbers of *F*. *prausnitzii* (*ρ* = -0.221, *P* = 0.092, **Fig 3A**). In contrast, a moderately positive correlation, though non-significant, was found between 24-h urinary ^52^Cr-EDTA/creatinine excretion and relative numbers of *Enterobacteriaceae* species (*ρ* = 0.202, *P* = 0.126, **Fig 3B**).

## Discussion

In this study, we show that increased urinary excretion of orally administered ^52^Cr-EDTA significantly correlates with elevated levels of fecal calprotectin in CD patients. Furthermore, CD patients with solely colonic disease activity show elevated ^52^Cr-EDTA/creatinine urinary excretion as opposed to CD patients having only an affected ileum. In addition, we observed an interesting trend of a negative association between ^52^Cr-EDTA-measured intestinal permeability and relative numbers of *F*. *prausnitzii*, whereas a trend of a positive association was observed between *Enterobacteriaceae* counts and urinary ^52^Cr-EDTA excretion. These correlations, though they lacked statistical significance, may suggest the possibility of a relationship between intestinal permeability and intestinal dysbiosis. Overall, our findings suggest that the measurement of urinary ^52^Cr-EDTA excretion might serve as novel approach for determining compromised intestinal permeability, as is characteristic for severe CD.

Chromium-EDTA has been widely used as marker for intestinal permeability, due to its high sensitivity and the stability of the Cr-EDTA complex. [35] However, most studies used the radioactive form ^51^Cr-EDTA that may have adverse effects due to radiation exposure. Here, we assessed the non-radioactive and inert ^52^Cr-EDTA to evaluate intestinal permeability in CD, in combination with quantifying urinary ^52^Cr by highly-sensitive ICP-MS. This method is particularly useful in detecting metal isotopes at very low concentrations in a safe, inexpensive and rigorously validated manner (Nexion 300X, Perkin Elmer, Medlon BV Enschede, The Netherlands). The currently applied ICP-MS method appeared to be highly sensitive in detecting ^52^Cr, with an internally determined lower limit of quantification (LLOQ) of 9.8 nmol/L. The average concentration of endogenous chrome in individuals in human blood and urine lies in the range of 0.1–0.5 μg/L (1.9–9.6 nmol/L), which falls below the LLOQ of ICP-MS and is thus negligibly small. For instance, the lowest individual value measured in our study cohort was 1352 nmol/L. [48]

This study assessed the ^52^Cr-EDTA intestinal permeability test in a relatively large study cohort of CD patients. Interestingly, the majority of these patients (70%) were in clinical remission according to the HBI, but still show a large variation in ^52^Cr-EDTA-measured intestinal permeability. Our study cohort is larger and less heterogeneous than all previous performed studies that have evaluated the potential application of Chromium-EDTA-measured intestinal permeability in IBD. [11,12,25–33] Patient participation within our study was high, due to the non-invasive and non-radioactive nature of this test. However, careful test monitoring was required since several factors could affect urinary excretion levels of ^52^Cr-EDTA, such as adherence to the experimental protocol and the duration of urine collection. [49] Also, in the present study cohort, no endoscopic disease activity data were available, which are preferentially used as representative of inflammatory disease activity in CD. Alternatively, we used the fecal calprotectin level as surrogate marker for CD disease activity. [19,20,50–52] Here, fecal calprotectin levels were directly related to ^52^Cr-EDTA-measured intestinal permeability, which has previously not been established.

Few studies specifically focused on the association between increased ^51^Cr-EDTA-measured intestinal permeability and inflammatory disease activity. [26,32,53] Hitherto, Berstad *et al*. found a significant correlation between 5-h urinary ^51^Cr-EDTA excretion and calprotectin levels in gut lavage fluid of IBD patients. [53] Another study observed an association between increased ^51^Cr-EDTA-measured intestinal permeability and serum levels of acute-phase reactants and ^111^In-labeled leukocyte scans. [32] In UC, a significant correlation between ^51^Cr-EDTA-measured intestinal permeability and endoscopic disease activity was demonstrated. [26] In CD, it has been shown that anti-TNF treatment attenuates mucosal inflammation and also improves intestinal integrity measured by orally administered ^51^Cr-EDTA. [54] Although the relationship between disease activity and Cr-EDTA-measured intestinal permeability seems to be well-established, studies show weak comparability because of various study designs, different intestinal permeability tracers and varying time intervals. Furthermore, it is difficult to clearly demonstrate this relationship since many factors are known to affect intestinal permeability, such as disease localization, surgical history, alcohol consumption, medication use, commensal bacteria and many dietary components. [55] As a consequence, the extent of this relationship remains inconclusive. In addition, all the previously mentioned studies are remarkably contradictory as to the primary intestinal location and degree of increased ^51^Cr-EDTA bowel passage. [25–29] In our study cohort, CD patients with exclusively colonic disease activity showed a higher median urinary ^52^Cr-EDTA/creatinine excretion as compared to patients with ileal involvement, though non-significantly. Similarly, we could not demonstrate any statistically significant differences in intestinal permeability between three different subgroups of disease localization (i.e. ileal, colonic or ileocolonic disease).

Until now, it remains unclear whether increased intestinal permeability is secondary to subclinical mucosal inflammation in CD or vice versa. [56,57] Accumulating evidence suggests that increased intestinal permeability may be the consequence of a genetically pre-existing epithelial barrier abnormality in certain individuals that predisposes them to the onset of intestinal inflammation. [58] Various studies have shown that a subset of clinically healthy first-degree relatives of Crohn’s disease patients show increased intestinal permeability as compared to healthy controls. [15,16] Moreover, it has been hypothesized that impaired intestinal permeability may already be apparent long before any mucosal inflammation is present and could be predictive of clinical relapse. In addition, another study demonstrated that in first-degree relatives of CD patients subclinical intestinal inflammation is present, reflected by an increased fecal calprotectin level. [59] In our study, we found a significant correlation between intestinal inflammatory disease activity (as represented by fecal calprotectin levels) and intestinal permeability (as measured by urinary ^52^Cr EDTA excretion). Overall, this indicates that ^52^Cr-EDTA-measured intestinal permeability might be particularly useful in defining the first stages of development of Crohn’s disease in a subgroup of unaffected, genetically susceptible individuals.

CD patients typically demonstrate a decreased gut microbiota diversity as compared to healthy individuals. [60] An important observation in CD is the reduction in the commensal anaerobic, butyrate-producing bacterium *F*. *prausnitzii*, as well as an increased number of *Enterobacteriaceae* (e.g. *Escherichia coli*). [3,6,61] In the present study, we observed a negative non-significant relationship between ^52^Cr-EDTA-measured intestinal permeability and the relative abundance of *F*. *prausnitzii*, while a positive non-significant relationship was observed between the relative abundance of *Enterobacteriaceae* and urinary ^52^Cr-EDTA excretion in CD patients. These interesting associations (though moderate and non-significant) support the existence of a link between the gut microbiota composition and the integrity of the intestinal barrier. Future studies are needed to further elucidate this relationship, for example through *in vitro* modeling of host-microbe interactions using bacteria-gut epithelial co-culture systems. [6] In this way, the influence of specific bacterial species on epithelial markers for intestinal permeability may give more fundamental insight into this complex association.

In conclusion, we show that a moderate positive correlation exists between ^52^Cr-EDTA-measured intestinal permeability and fecal calprotectin levels. In addition, we demonstrate interesting, though non-significant correlations between intestinal permeability and two key gut bacterial species, suggesting the possibility of a relationship between dysbiosis and permeability. Future studies should primarily focus on the utility of orally administered ^52^Cr-EDTA as measure of intestinal permeability in relation to endoscopic disease activity and its potential role as predictor of CD disease exacerbations.

## Supporting information

S1 Fig Fluorescence in situ hybridization (FISH) experiments quantify the absolute and relative amounts of selected bacteria in fecal samples. Paraformaldehyde-fixed fecal samples were hybridized with specific fluorescent oligonucleotide probes and visualized with an Olympus BH2 epifluorescence microscope. Images are shown as examples and are taken from random samples not related to each other. **(A)** Epifluorescent images of a hybridization with a Rhodamine-labeled Eub338 probe specific for almost all bacteria at a 1600 x dilution of a fecal patient sample. **(B)** Hybridization with a FITC-labeled *Faecalibacterium prausnitzii*-specific probe Fprau645 of a 160 x diluted fecal sample. **(C)** Hybridization with the Fprau645 probe at 40 x dilution of the fecal sample. **(D)** Hybridization with the CY3-labeled *Enterobacteriaceae*-specific probe Ec1531 at a 40x dilution of the fecal sample. Bar, 20 μm.
(TIF)

S1 Table Median numbers of *F*. *prausnitzii* and *Enterobacteriaceae* per gram of feces and their relative abundances (%) to total microbiota (Eub338). Data are shown for the total study cohort (*n* = 60), and for subgroups of CD patients with below- and above-median urinary ^52^Cr-EDTA/creatinine excretion. Data are presented as median (IQR).
(DOCX)
