# Supplementary figures and images for: Assessing intestinal permeability in Crohn’s disease patients using orally administered 52Cr-EDTA
Source: PLoS One. 2019 Feb 7;14(2):e0211973. doi: 10.1371/journal.pone.0211973 (PMC6366711; doi:10.1371/journal.pone.0211973)

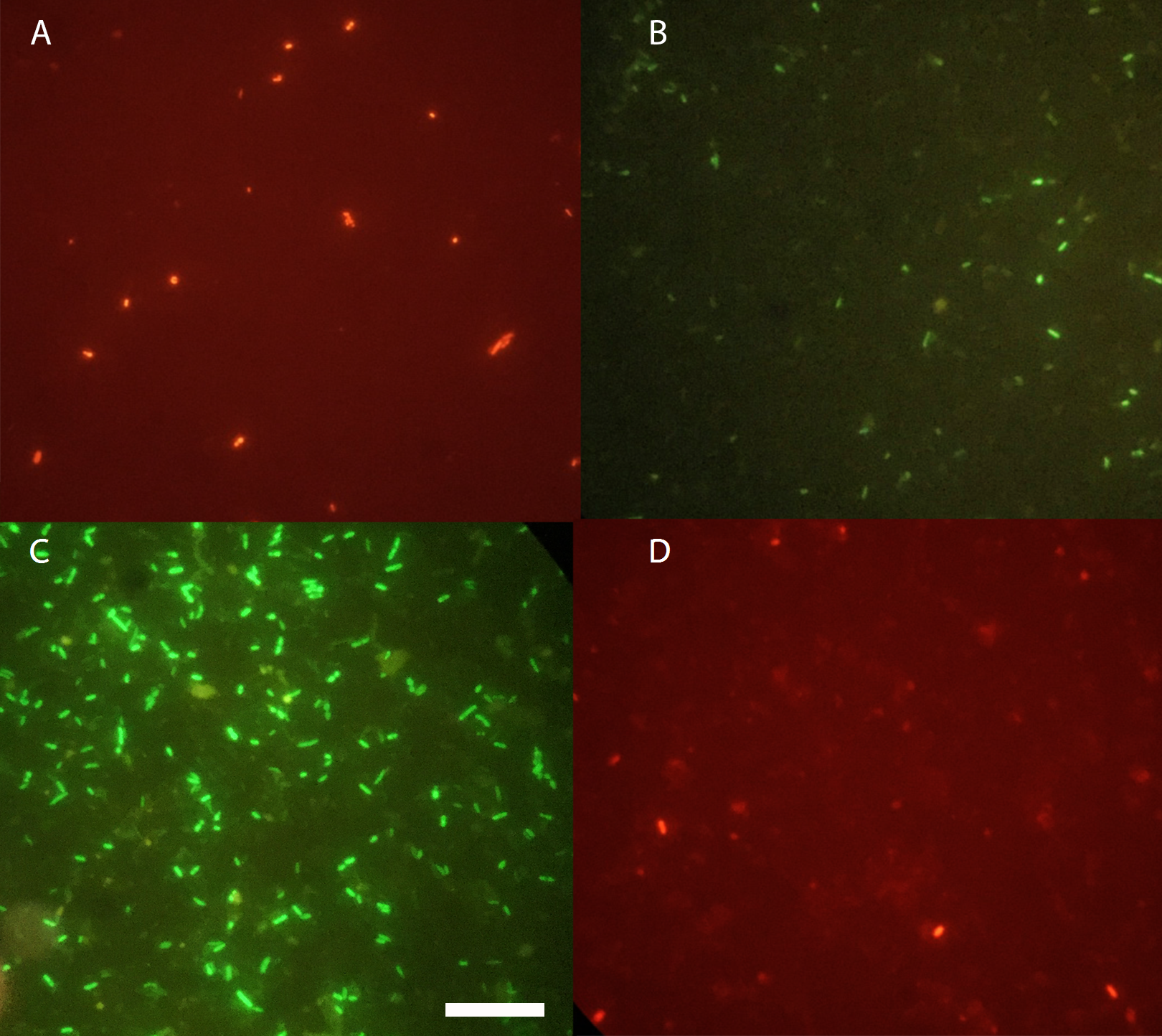

Supplement: S1 Fig — Paraformaldehyde-fixed fecal samples were hybridized with specific fluorescent oligonucleotide probes and visualized with an Olympus BH2 epifluorescence microscope. Images are shown as examples and are taken from random samples not related to each other. (A) Epifluorescent images of a hybridization with a Rhodamine-labeled Eub338 probe specific for almost all bacteria at a 1600 x dilution of a fecal patient sample. (B) Hybridization with a FITC-labeled Faecalibacterium prausnitzii-specific probe Fprau645 of a 160 x diluted fecal sample. (C) Hybridization with the Fprau645 probe at 40 x dilution of the fecal sample. (D) Hybridization with the CY3-labeled Enterobacteriaceae-specific probe Ec1531 at a 40x dilution of the fecal sample. Bar, 20 μm. (TIF) [file pone.0211973.s001.tif]
