# Supplementary material for: Assessing intestinal permeability in Crohn’s disease patients using orally administered 52Cr-EDTA
Source: PLoS One. 2019 Feb 7;14(2):e0211973. doi: 10.1371/journal.pone.0211973 (PMC6366711; doi:10.1371/journal.pone.0211973)
Supplement: S1 Table — Data are shown for the total study cohort (n = 60), and for subgroups of CD patients with below- and above-median urinary 52Cr-EDTA/creatinine excretion. Data are presented as median (IQR). (DOCX) [file pone.0211973.s002.docx]

| Target | **Probe** | **Bacteria/gram of feces** | Relative abundance (%) |
| --- | --- | --- | --- |
| *Total study cohort (n = 60)* | | | |
| *F. prausnitzii* | Fprau645 | 7.05 x 10^8^ (1.10 x 10^8^ – 9.91 x 10^8^) | 5.62 (2.68 – 9.37) |
| *Enterobacteriaceae* | Ec1531 | 6.67 x 10^6^ (1.78 x 10^6^ – 5.47 x 10^7^) | 0.10 (0.02 – 1.13) |
| *Below-median ^52^Cr-EDTA/creatinine excretion (< 678.6 µmol/mol) (n = 30)* | | | |
| *F. prausnitzii* | Fprau645 | 5.97 x 10^8^ (1.37 x 10^8^ – 9.42 x 10^8^) | 5.62 (2.83 – 10.1) |
| *Enterobacteriaceae* | Ec1531 | 4.45 x 10^6^ (1.33 x 10^6^ – 4.78 x 10^7^) | 0.06 (0.01 – 0.75) |
| *Above-median ^52^Cr-EDTA/creatinine excretion (> 678.6 µmol/mol) (n = 30)* | | | |
| *F. prausnitzii* | Fprau645 | 4.89 x 10^8^ (8.06 x 10^7^ – 1.40 x 10^9^) | 5.16 (2.14 – 9.60) |
| *Enterobacteriaceae* | Ec1531 | 1.74 x 10^7^ (3.45 x 10^6^ – 1.17 x 10^8^) | 0.12 (0.02 – 2.71) |
